# Transplantation of human neural stem/progenitor cells overexpressing galectin-1 improves functional recovery from focal brain ischemia in the mongolian gerbil

**DOI:** 10.1186/1756-6606-4-35

**Published:** 2011-09-27

**Authors:** Junichi Yamane, Satoru Ishibashi, Masanori Sakaguchi, Toshihiko Kuroiwa, Yonehiro Kanemura, Masaya Nakamura, Hiroyuki Miyoshi, Kazunobu Sawamoto, Yoshiaki Toyama, Hidehiro Mizusawa, Hideyuki Okano

**Affiliations:** 1 Department of Physiology, Keio University School of Medicine, Tokyo, Japan; 2 Department of Orthopaedic Surgery, Keio University School of Medicine, Tokyo, Japan; 3 Department of Orthopaedic Surgery, Tokyo Dental College Ichikawa General Hospital, Chiba, Japan; 4 Department of Neurology and Neurological Science, Graduate School of Medicine, Tokyo Medical and Dental University, Tokyo, Japan; 5 Department of Clinical Laboratory, Namegata District General Hospital, Ibaraki, Japan; 6 Division of Regenerative Medicine, Institute for Clinical Research, Osaka National Hospital, National Hospital Organization, Osaka, Japan; 7 Department of Neurosurgery, Osaka National Hospital, National Hospital Organization, Osaka, Japan; 8 Subteam for Manipulation of Cell Fate, RIKEN BioResource Center, Ibaraki, Japan; 9 Bridgestone Laboratory of Developmental and Regenerative Neurobiology, Keio University School of Medicine, Tokyo, Japan; 10 Department of Developmental and Regenerative Biology, Nagoya City University Graduate School of Medical Sciences, Aichi, Japan

## Abstract

Transplantation of human neural stem/progenitor cells (hNSPCs) is a promising method to regenerate tissue from damage and recover function in various neurological diseases including brain ischemia. Galectin-1(Gal1) is a lectin that is expressed in damaged brain areas after ischemia. Here, we characterized the detailed Gal1 expression pattern in an animal model of brain ischemia. After brain ischemia, Gal1 was expressed in reactive astrocytes within and around the infarcted region, and its expression diminished over time. Previously, we showed that infusion of human Gal1 protein (hGal1) resulted in functional recovery after brain ischemia but failed to reduce the volume of the ischemic region. This prompted us to examine whether the combination of hNSPCs-transplantation and stable delivery of hGal1 around the ischemic region could reduce the ischemic volume and promote better functional recovery after brain ischemia. In this study, we transplanted hNSPCs that stably overexpressed hGal1 (hGal1-hNSPCs) in a model of unilateral focal brain ischemia using Mongolian gerbils. Indeed, we found that transplantation of hGal1-hNSPCs both reduced the ischemic volume and improved deficits in motor function after brain ischemia to a greater extent than the transplantation of hNSPCs alone. This study provides evidence for a potential application of hGal1 with hNSPCs-transplantation in the treatment of brain ischemia.

## Background

Stem cell-based therapies have been performed in various clinical settings, although many lack scientific evidence of their effectiveness [1]. Among stem cell-based therapies, transplantation of human neural stem/progenitor cells (hNSPCs) is relatively well substantiated by peer-reviewed literatures [2-8]. One reason underlying the relative success of hNSPCs-transplantation is its low occurrence of tumor formation, which is a clear advantage compared with transplantation of embryonic stem cells or their derivatives [9]. Therefore, we have been examining hNSPCs-transplantation in various preclinical animal models and have shown that hNSPCs-transplantation enhances functional recovery following brain ischemia [10] and spinal cord injury (SCI) [11].

Brain ischemia, which is caused by occlusion of a cerebral artery, leads to focal tissue loss and death of multiple neuronal cell types within and around the ischemic region. Patients with brain ischemia exhibit persistent motor, sensory or cognitive impairments, which have devastating effects on their quality of life. Apart from acute thrombolysis, which can be used in only a minority of cases, there is still no effective treatment to promote functional recovery after brain ischemia.

hNSPCs can generate all principle cell types (i.e., neurons, astrocytes and oligodendrocytes) in the brain and therefore have great therapeutic potential in severe neurological diseases, including brain ischemia [6,12], which induce death of various cell types [13,14]. hNSPCs can be propagated in large quantities for long-term without a notable loss of the ability to proliferate and differentiate [15]. Therefore, cultured hNSPCs are a promising cell source to treat brain diseases.

We previously showed that transplantation of cultured hNSPCs reduced infarct volume and improved functional prognosis in a rodent model of brain ischemia [10]. In the damaged brains of the model animals, hNSPCs differentiated into mature neurons within the ischemic region, and some of those new-born neurons were incorporated into the host neural circuitry [10]. In SCI model mice, grafted hNSPCs differentiated into oligodendrocytes and contributed to re-myelination of host neuronal axons [5]. Another possible mechanism of the therapeutic effects of hNSPCs-transplantation is their trophic actions. It has been suggested that grafted hNSPCs release molecules which exert neuro-protective roles or reduce inflammation [10].

Galectin-1(Gal1) is expressed around infarcted tissue after brain ischemia [10,16]. Gal1 is a soluble lectin that binds to lactosamine-rich carbohydrate moieties on various molecules [17]. Although its binding partner in the mammalian brain seems relatively limited [18], Gal1 is expressed in adult NSCs in the subventricular zone (SVZ) of the lateral ventricles (LV) [19,20] and the dentate gyrus (DG) of the hippocampus [21]. We showed that infusion of human recombinant Gal-1 protein (hGal1) enhanced functional recovery in a rodent model of brain ischemia [20] but failed to reduce the volume of the infarcted area [20]. Because hNSPCs-transplantation was effective in reducing infarct volume after brain ischemia [10], we hypothesized that the combination of hNSPCs-transplantation and continuous delivery of Gal1 at the same time would reduce the volume of the infarcted area and improve functional recovery to a greater extent than hNSPCs-transplantation alone. Indeed, we previously showed that transplantation of hNSPCs overexpressing hGal1 (hGal1-hNSPCs) led to a better functional outcome than transplantation of hNSPCs alone in a non-human primate model of SCI [22].

In the present study, we analyzed the time course of intrinsic Gal1 expression after brain ischemia. Next, we examined the therapeutic effect of transplantation of hGal1-hNSPCs compared with hNSPCs alone, and found that hGal1-hNSPCs reduced the infarct volume and resulted in better functional recovery after brain ischemia.

## Methods

### Culture of hNSPCs

This study was carried out in accordance with the principles of the Helsinki Declaration, and the Japan Society of Obstetrics and Gynecology. Approval to use human fetal neural tissues was obtained from the ethical committees of both Osaka National Hospital and Keio University. Written informed consent was obtained from all parents through routine legal terminations performed at Osaka National Hospital.

hNSPCs (oh-NSC-3-fb) were isolated from fetal forebrain tissues (10 gestational weeks [GW]) and propagated using a defined neural progenitor cell basal medium (NPBM; Clonetics)-based medium supplement with human recombinant (hr-) basic fibroblast growth factors-2 (FGF-2, 20 ng/ml; R&D), hr-epidermal growth factor (EGF, 20 ng/ml; R&D), hr-leukemia inhibitory factor (LIF, 10 ng/ml; Chemicon), and GA-100 (5 μg/ml gentamicin sulfate, 5 ng/ml amphotericin B; Clonetics) as described previously [15,23].

### Lentiviral transduction of hNSPCs

hNSPCs that had undergone more than 10 passages were dissociated into single cells 2 hr before being infected. The concentrated viruses were added to the culture medium to infect the hNSPCs [multiplicity of infection (MOI) = 5]. Two weeks later, neurospheres were formed from the dissociated hNSPCs and were passaged. The efficiency of the transduction was measured by GFP expression with a FACS Calibur (Beckton Dickinson). hNSPCs with an transduction efficiency of greater than 80% were used for transplantation. The third-generation self-inactivating HIV-1-based lentiviral vector pCSII-EF-MCS-IRES2-GFP [24] was used for the transduction. Two types of lentivirus-transduced hNSPCs were prepared: hGal1-hNSPCs, which were hNSPCs infected with the human Gal1 IRES GFP virus; and hNSPCs, which were hNSPCs infected with the IRES GFP virus.

### Animals

Animal experiments were approved by the Animal Experiment Committee of Tokyo Medical and Dental University. Thirty-six male Mongolian gerbils (aged 16-22 weeks and weighing 60-72 g) were housed in groups (3-4 per cage) and maintained on a 14:10-hr light:dark cycle with unlimited access to food and water.

### Focal Ischemic Surgery

To induce brain ischemia, animals were anesthetized with 2% isoflurane. The left common carotid artery was occluded with a mini vascular clip for 10 min, after which animals were allowed to recover from anesthesia. During the carotid artery occlusion, stroke symptoms were evaluated using a stroke index (SI)[25]. Animals manifesting a SI of more than 10 were selected as 'post-ischemic animals' [25]. In post-ischemic animals, a second 10-min period of ischemia was similarly induced 5 hr later.

### Transplantation Surgery

Four days after ischemic surgery, post-ischemic animals were randomly assigned to hNSPCs and hGal1-hNSPCs groups, and the hNSPCs-suspension was transplanted into the caudate nucleus of the lesioned hemisphere. Animals were anesthetized with 2% isoflurane and placed in a stereotaxic frame. A hole was drilled in the left side of the skull to allow the penetration of a 10 μL Hamilton syringe at the following coordinates (mm) relative to bregma: anterior 1.0 mm; lateral 3.0 mm; and ventral 3.0 mm. A 3 μL aliquot of hNSPCs-suspension (50,000 cells) was infused over 2 min, and the syringe was left in place for an additional 2 min to allow diffusion from the tip. All animals received cyclosporine A (10 mg/kg intramuscularly; Wako) 24 hr before transplantation and three times per week for 4 weeks thereafter.

### Histological Analyses

At the end of the observation period, animals were anesthetized deeply with diethyl ether, sacrificed, and fixed by perfusion with 4% paraformaldehyde. The post-fixed brains were cut into 50-μm coronal sections using a vibratome. The coronal sections were stained with Nissl for detection and calculation of the ischemic injury area. The area of infarction was measured using the MCID system [22,26,27](InterFocus Imaging), using automatic tiling and area size quantification options at the same exposure time and threshold settings, and then compared between hNSPCs- and hGal1-hNSPCs-transplanted groups. The total infarct area (indirect lesion volume) of the ipsilateral hemisphere was calculated as a percentage of the volume of the contralateral hemisphere, as reported previously [28].

hNSPCs grafted into the injured brain were identified using an anti-GFP antibody (1:200; MBL), and their phenotypes were examined by immunostaining for the cell-type-specific markers anti-NeuN (1:100; Sigma) and anti-GFAP (1:200; Dako). Grafted NSCs co-labeled with GFP and cell-type-specific markers were detected with a confocal microscope equipped with an argon-krypton laser (LSM510; Zeiss) and a fluorescence microscope (Axioskop 2 Plus; Zeiss).

### BrdU labeling

To label S-phase cells, the thymidine analog 5-bromo-2'-deoxyuridine-5'-monophosphate (BrdU) was administered intraperitoneally (50 mg/kg; Sigma). On day 3 after the infarction, two injections (6 h apart) of BrdU were given. 24 hours later, animals were euthanized by transcardiac perfusion. This allowed us to measure the number of cells that incorporated BrdU during a 24-h period and provided an index of the rate of cell birth at a specific time point after ischemia.

### Behavioral Tests

Animals were subjected to a series of behavioral tests within 30 days after focal ischemia. The researcher conducting the behavioral testing and scoring was blind to the experimental conditions. All animals were videotaped during behavioral tests. The elevated body swing test (EBST) was used to evaluate asymmetric motor behavior [29]. Animals were held by the base of the tail and elevated about 10 cm from the tabletop. The direction of body swing, defined as an upper body turn of 10 degrees to either side, was recorded for 1 min during each of the three trials per day. The number of left and right turns was counted, and the percentage of turns made to the side contralateral to the damaged hemisphere (% right-biased swing) was determined. The bilateral asymmetry test (BAT) is a test of unilateral sensory dysfunction [30]. Two small pieces of adhesive-backed paper dots were used as bilateral tactile stimuli occupying the distal-radial region on the wrist of each forelimb. The time, to a maximum of 3 min, that it took each animal to remove each stimulus from the forelimb (removal time) was recorded in three trials per day. The T-maze spontaneous alternation task is a method to test spatial cognitive function [31]. Animals were allowed to alternate between the left and right goal arms of a T-shaped maze throughout a 15-trial continuous alternation session. Once they had entered a particular goal arm, a door was lowered to block entry to the opposite arm. The door was reopened only after animals returned to the start arm, thus allowing a new alternation trial to be started. Their behavior was traced with a video-tracking system (PanLab, Barcelona, Spain). The spontaneous alternation rate was calculated as the ratio between the alternating choices and total number of choices.

### Statistical Analysis

Unpaired t-tests (for two groups) or repeated measures ANOVAs were used to detect differences between groups in behavioral analyses and histological quantifications.

## Results

### Gal1 expression in brain after focal brain ischemia

To study the expression of Gal1 after brain ischemia, we utilized an animal model of brain ischemia established in our group [10,20,32,33]. Consistent with our previous studies, induction of transient focal ischemia in Mongolian gerbils resulted in widespread ischemic lesions in the cerebral cortex, caudate nucleus, thalamus and hippocampus (Additional file 1, Fig. S1). Sensorimotor and cognitive dysfunctions were observed after the induction of ischemia as previously reported [10,20,32,33].

**Figure 1:**
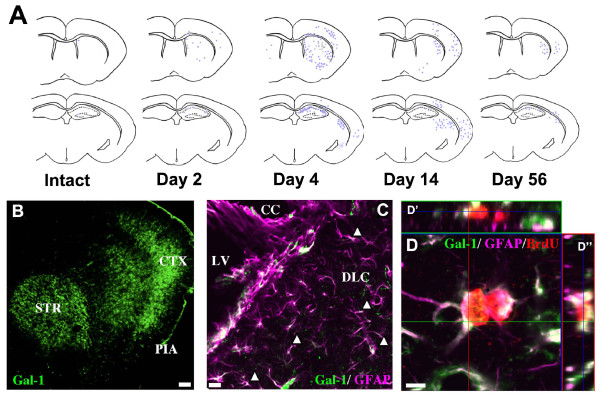
**Characterization of Gal1 expression after brain ischemia**. **A: **Diagram of intrinsic Gal1 expression before and after brain ischemia. Two representative coordinates of coronal sections, where corresponding functional tests were performed later, were chosen for analysis. Gross anatomy of each brain section was delineated. The density of purple dots corresponds to the intensity and extent of signals from immunohistochemistry using a Gal1-specific antibody. As time passed following ischemia, intrinsic expression of Gal1 was temporarily elevated and then gradually decreased (left to right). **B**. A representative image of Gal1-immunohistochemisty at day 4 after brain ischemia. (Scale bar: 100 μm). STR, striatum; CTX, cortex; PIA, pia mater. **C**. Gal1 (green) was expressed in GFAP-positive (purple) astrocytes in the SVZ. Note that Gal1 was also expressed outside of the SVZ (arrowheads) after brain ischemia. (Scale bar: 25 μm). LV, lateral ventricle; CC, corpus callosum; DLC, dorso-lateral corner. **D**. Most Gal1-positive (green) cells outside the SVZ were proliferating (BrdU-positive, red) astrocytes (GFAP-positive, purple). (Scale bar: 10 μm). D' and D", z-stack images (image of the plane perpendicular to that shown in D).

To study the expression of Gal1 after ischemia, immunohistochemical analyses were performed using Gal1-specific antibody which gave rise to no staining on brain section from *galectin-1 *null mutant mice [19,21]. In the normal adult Monglian gerbil brain, expression of Gal1 was rarely found outside of the SVZ of the LVs and the DG of the hippocampus except in a few neurons and blood vessels throughout the brain (Additional file 2, Fig. S2), which is comparable to the pattern of Gal1 expression in the adult mouse brain [19,21]. Four days after brain ischemia, Gal1 expression was widely induced in the core and around the ischemic region (Figure 1A, left 1B). At later time points, Gal1 expression was gradually diminished (Figure 1A, *middle *and right). The vast majority of Gal1-positive cells were GFAP-positive astrocytes in the core and around the ischemic regions (Figure 1C), and no more than 1% of Gal1 expressing cells were NeuN-positive neurons (Additional file 3, Fig. S3). Interestingly, ischemia-induced Gal1 expression was found mostly in BrdU-positive proliferating reactive astrocytes outside the SVZ (Figure 1D).

### Transplantation of hGal1-hNSPCs enhanced the recovery of tissue damage after brain ischemia

**Figure 2:**
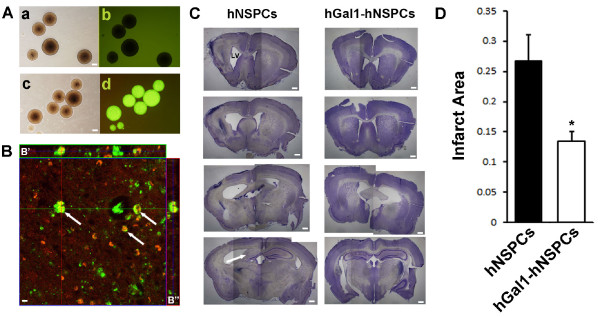
**Transplantation of hGal1-hNSPCs reduced infarct volume after brain ischemia to a greater extent than hNSPCs alone**. **A: **hNSPCs were cultured *in vitro *for more than 10 weeks (a, transmitted light image, b, GFP-filtered image under fluorescent light) and induced to express hGal1 using lentivirus (c, transmitted light image, d, GFP-filtered image under fluorescent light). (Scale bar: 50 μm) **B**. After transplantation, hNSPCs-derived cells (Green) survived, with some expressing a mature neuronal marker, NeuN (Red)(Arrows). (Scale bar: 10 μm). B' and B", z-stack images. **C**. Representative images of Nissl staining of hNSPCs- or hGal1-hNSPCs-transplanted gerbil brains. (Scale bar: 500 μm). Ischemia was induced unilaterally (on the left side). Note the enlargement of the LV and marked neuronal loss particularly evident in neuronal-dense areas such as the hippocampus (arrow) in the hNSPCs group, which were recovered the in hGal1-hNSPCs group. LV, lateral ventricle. **D**. The volume of the ischemic area was significantly reduced in the hGal1-hNSPCs group (n = 7) compared to the hNSPCs group (n = 6)(*p < 0.05).

To examine the combinational effects of hNSPCs-transplantation and hGal1 delivery, hGal1 was permanently expressed in hNSPCs using a lentivirus vector (Figure 2A, Additional file 4 Fig. S4) as previously described [22]. Since the amount of intrinsic Gal1 expression peaked between 4 days and 2 weeks after brain ischemia (Figure 1A), we hypothesized that the therapeutic effect of hGal1 would be maximal around this time point. Therefore, hGal1-hNSPCs were transplanted around the ischemic region 4 days after brain ischemia. Substantial numbers of transplanted cells survived in both hNSPCs and hGal1-hNSPCs groups, similar to previous reports [10]. NeuN, a nuclear marker of mature neurons, and GFP, the marker of transplanted hNSPCs, were utilized to examine the maturation of transplanted cells. Similar ratios and distributions of NeuN/GFP-double positive cells were detected after transplantation in both groups, suggesting that some hNSPCs differentiated into mature neurons after transplantation (Figure 2B). Although we could not determine the exact number of surviving cells, mainly because they were spread widely throughout the brain, there was no apparent difference in the number of transplanted cells between the hGal1-hNSPCs and hNSPCs groups. Nissl staining was performed to analyze the extent of tissue damage 30 days after transplantation. Interestingly, the volume of the ischemic region was significantly reduced in the hGal1-hNSPCs group compared to the hNSPCs group (Figure 2C, D, planned comparison *t*(9) = 3, *p *= 0.01, also see additional file 5, Figure S5). This suggests that the therapeutic effect of hNSPCs-transplantation can be augmented by the overexpression of hGal1.

### Transplantation of hGal1-hNSPCs resulted in better functional recovery

**Figure 3:**
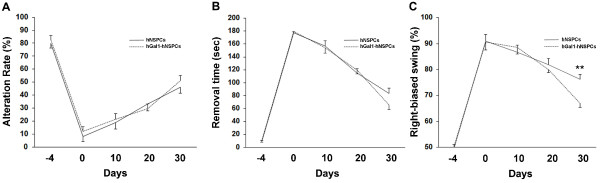
**Transplantation of hGal1-hNSPCs promoted better functional recovery after brain ischemia than hNSPCs alone**. **A: **Cognitive function was analyzed by the T-maze test before and after the induction of ischemia. Before ischemia, gerbils showed around a 80% preference for left choices (day - 4). After ischemia, both groups showed equivalent recovery. Black line: hNSPCs (n = 6), dotted line: hGal1-hNSPCs (n = 7). **B**. Sensory function was examined using the BAT. Both group showed similar recovery across the observation period. The hGal1-hNSPCs group (dotted line: n = 7) showed a tendency toward better recovery (reduced time to remove tape attached to forelimbs) on day 30 compared to the hNSPCs group (black line: n = 6). **C**. EBST was performed to examine motor function. After brain ischemia, gerbils showed a tendency to swing their bodies towards the non-ischemic side (day 0). Motor function gradually improved in both groups after transplantation. The hGal1-hNSPCs group (dotted line: n = 7) showed better recovery overall compared to the hNSPCs group (black line: n = 6) (**p < 0.005).

Finally, we compared the functional outcome of the transplantation of hGal1-hNSPCs or hNSPCs. We carried out 3 behavioral tests: the elevated body swing test (EBST) for examining motor function, the bilateral asymmetry test (BAT) for somatosensory function, and the T-maze test for cognitive function. All of these tests have previously been used to evaluate functional outcome after ischemia, and we reproduced previous results across the three different tests over the time period after brain ischemia (Figure 3)[10,20,32,33]. In the T-maze test, both groups showed recovery across the observation period, with no difference between groups (Figure 3A, time × group ANOVA, interaction *F*(4, 44) = 0.52, *p *= 0.7, main effect of time *F*(4, 44) = 137, *p *< 0.001). In the BAT, both groups showed recovery after transplantation (Figure 3B, time × group ANOVA, interaction *F*(4, 44) = 1.3, *p *= 0.3, main effect of time *F*(4, 44) = 301, *p *< 0.001), with a tendency toward greater improvement in the hGal1-hNSPCs group compared to the hNSPCs group on day 30 (planned comparison, *t*(11) = 2, *p *= 0.06). This suggests that hGal1-hNSPCs improve cognitive and sensory function to a similar extent as hNSPCs. Interestingly, in the EBST, the hGal1-hNSPCs group showed better improvement after transplantation compared to the hNSPCs group (Figure 3C, time × group ANOVA, interaction *F*(4, 44) = 6.4, *p *= 0.05, main effect of time *F*(4, 44) = 141, *p *< 0.001, planned comparison on day 30 *t*(11) = 4, *p *= 0.002), suggesting that hGal1-hNSPCs-transplantation results in better recovery of motor function after brain ischemia.

## Discussion

In this study, we showed that Gal1 expression was induced in proliferating reactive astrocytes after brain ischemia and gradually decreased over time. The transplantation of hGal1-hNSPCs after brain ischemia resulted in reduced infarcted volume and better recovery of motor function compared to transplantation of hNSPCs alone. These findings suggest a potential use of hGal1 in combination with transplantation of hNSPCs in the treatment of brain ischemia.

Although transplantation of hGal1-hNSPCs reduced ischemic volume and promoted functional recovery from brain ischemia, the mechanisms underlying these effects are unclear. It is reasonable to consider that the extent of functional recovery is correlated to the size of the infarcted region after brain ischemia as shown by this study and others [10,34]. It is possible that the reduced volume of ischemic region was caused by either an increase in the number of transplanted cells after transplantation or a preservation of the otherwise-dying host tissue. Since i) our previous reports showed that hGal1 overexpression neither promoted proliferation or survival nor changed the direction of differentiation (Appendix One) of human neurosphere-derived NSPCs [22] and ii) we observed no apparent differences in the survival of transplanted cells by overexpression of hGal1, which agrees with previous studies [22], the reduced volume of the ischemic region could a result from preservation of the host tissue through two possible mechanisms.

First, trophic factors, which may be released from hNSPCs, could preserve the damaged tissue [13,35]. Therefore, it is possible that hNSPCs might be induced to express greater amounts and varieties of these trophic factors (e.g., BDNF [16,36]), by the over-expression of hGal1. This possibility could be further investigated, for example, by microarray analysis in future experiments. Second, prolonged expression of hGal-1 released from hGal1-hNSPCs might have altered the proliferation and/or migration of reactive astrocytes, thereby preventing further tissue damage after brain ischemia. Considering that i) reactive astrocytes could play a crucial role in preventing the enlargement of the infarcted region after brain ischemia [34,37], ii) reactive astrocytes express Gal1 (Figure 1B) [16], iii) Gal1 regulates the proliferation of reactive astrocytes [16], iv) soluble Gal1 binds to β1 integrin [18,38], which regulates the migration of astrocytes [39,40], hGal-1 could influence the proliferation and/or migration of reactive astrocytes after brain ischemia, resulting in a reduction in the size of the ischemic region.

Furthermore, there may be other factors that promote functional improvement independent of the volume of the ischemic region. For these, it is important to consider the function of hGal-1 in promoting neurite outgrowth [22,41], possibly through enhancing the binding of neurites to β1 integrin [18,38]. After brain ischemia, neurite outgrowth is prevented by inflammatory cytokines and gliosis within and around the ischemic region [42,43]. Indeed, after stroke, promotion of neurite outgrowth improved neurological outcome [44]. Also, in our SCI model, it was suggested that the therapeutic effect of the transplantation of hGal-1-hNSPCs was due to increases in neurite outgrowth [22,41]. Thus, the overexpression of hGal1 in hNSPCs might increase neurite outgrowh of graft and/or host neurons in the ischemic brain, which could contribute to better functional recovery. These possibilities should be verified in future studies.

Previously, we showed that infusion of hGal1 protein improved recovery from motor and sensory (but not from cognitive) deficits after brain ischemia [20]. In this study, we did not see significant differences between the hGal1-hNSPCs-transplanted group and the naïve hNSPCs-transplanted group in the rate of recovery from sensory deficits. This raises the possibility that transplantation of hNSPCs alone is sufficient to promote recovery from sensory deficits caused by ischemia [20] and is not augmented by hGal1 overexpression. Indeed, in the current study, the hGal1-hNSPCs group showed only marginal improvement in the recovery from sensory deficit compared with hNSPCs group (Figure 3B). This underscores the observation that hGal1 has an additional benefit in the recovery of motor function beyond the effect of hNSPCs transplantation alone.

Considering that Gal1 is expressed endogenously in damaged tissue of various neurological diseases, and application of hGal1 improves symptoms of those diseases [16,22,41,45-47], hGal1 is a promising therapeutic agent. This study provides an alternative method to apply hGal1 the treatment of brain ischemia with transplantation of hNSPCs or alternative cells sources such as Nestin-expressing hair follicle stem cells [48-51].

## List of Abbreviations used

Gal1, NSCs, hNSPCs, SVZ, LV, BrdU, GFAP, EBST, BAT

## Competing interests

The authors declare that they have no competing interests.

## Authors' contributions

Author contributions: JY, SI, MS, NM, KS and HO designed the research. JY, SI, MS, TK, MH and YK performed the research, JY, SI and MS analyzed data, JY, SI, MS, TK, KS, MN, YT and HO prepared the manuscript. All authors read and approved the final manuscript.

## Appendix One

### hGal1 overexpression did not change the direction of differentiation into neurons and astrocytes

After 1 week of transduction of the external genes (i.e., GFP or hGal1/GFP), the neurospheres were dissociated into single cells and cultured onto poly-L-lysine coated coverglass. Seven days later, the cells were fixed and immunostained using cell type specific markers GFAP and Tuj-1 (a neuronal marker). The percentage of cells positive for each marker were as follows (mean ± SEM): GFAP-positive (hNSPCs, 22.3% ± 4.3, hGal1-hNSPCs, 24.6% ± 4.9), Tuj1-positive (hNSPCs, 36.9% ± 2.43, hGal1-hNSPCs, 37.9% ± 1.3).

## Supplementary Material

Additional file 1 **Figure S1**. Reproducible focal brain ischemia model in Mongolian gerbil. Photographic display of representative H&E-stained coronal brain sections taken from gerbils 4 weeks after unilateral carotid artery occlusion. Notice the enlargement of the LV and marked cell loss (arrows) on the side of ischemia (left). (Scale bar: 500 μm). LV, lateral ventricle. Click here for file

Additional file 2 **Figure S2**. Intrinsic Gal1 expression in brain of normal adult Mongolian gerbil. Gal1 was visualized by immunohistochemistry using DAB in coronal sections from normal adult gerbils. The signal (arrows) was detected around the SVZ (A, B: enlarged picture of the boxed part of A) of the adult gerbil brain. A few Gal1-positive cells were also detected outside the SVZ (arrowheads in B and C). (Scale bar: A, 50 μm, B, C, 15 μm). LV, lateral ventricle; Str, striatum; WM, white matter; CTX, cortex. Click here for file

Additional file 3 **Figure S3**. The vast majority of Gal1-positive cells around the ischemic region were NeuN-negative. Representative image of Gal1(Red) and NeuN (Green) double-immunostaining around the ischemic region after brain ischemia (2 weeks after occlusion). (Scale bar: 25 μm). Click here for file

Additional file 4 **Figure S4**. Gal1 is expressed in the supernatant of hGal1-hNSPCs and hNSPCs cultures. The supernatant was isolated from hGal1-hNSPCs and hNSPCs cultures (n = 3 each), condensed, and then total volume of 300 μg protein from each culture was applied for western blot analysis using anti-hGal1-specific antibody. hGal1 (14.5 kDa) was detected in all of the supernatants. Note that the amount of hGal1 was increased in hGal1-hNSPCs culture compared with the hNSPCs culture. Click here for file

Additional file 5 **Figure S5**. Comparison of infarct area. Infarct area was calculated in the brains of no-transplantation, hNSCs, hGal1-hNSCs and intact groups. Click here for file
