# Supplementary figures and images for: Transplantation of human neural stem/progenitor cells overexpressing galectin-1 improves functional recovery from focal brain ischemia in the mongolian gerbil
Source: Mol Brain. 2011 Sep 27;4:35. doi: 10.1186/1756-6606-4-35 (PMC3215926; doi:10.1186/1756-6606-4-35)

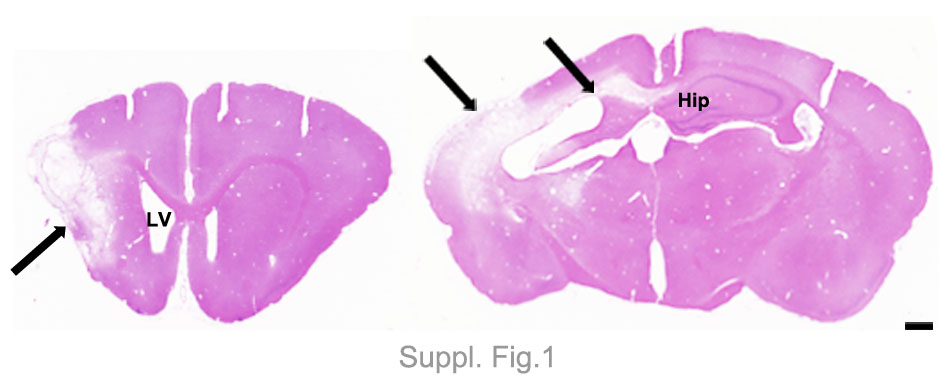

Supplement: Additional file 1 — Figure S1. Reproducible focal brain ischemia model in Mongolian gerbil. Photographic display of representative H&E-stained coronal brain sections taken from gerbils 4 weeks after unilateral carotid artery occlusion. Notice the enlargement of the LV and marked cell loss (arrows) on the side of ischemia (left). (Scale bar: 500 μm). LV, lateral ventricle. [file 1756-6606-4-35-S1.JPEG]

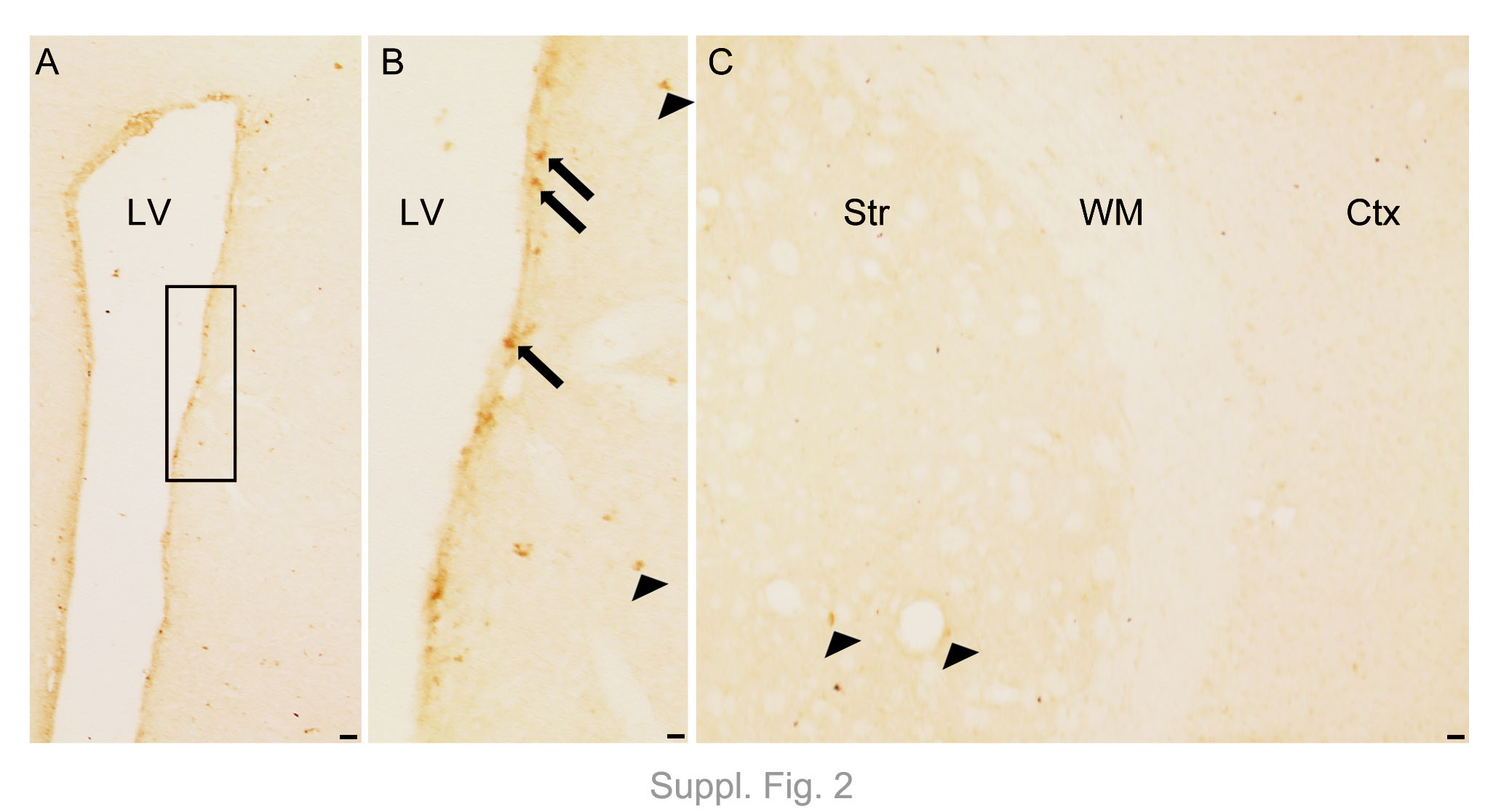

Supplement: Additional file 2 — Figure S2. Intrinsic Gal1 expression in brain of normal adult Mongolian gerbil. Gal1 was visualized by immunohistochemistry using DAB in coronal sections from normal adult gerbils. The signal (arrows) was detected around the SVZ (A, B: enlarged picture of the boxed part of A) of the adult gerbil brain. A few Gal1-positive cells were also detected outside the SVZ (arrowheads in B and C). (Scale bar: A, 50 μm, B, C, 15 μm). LV, lateral ventricle; Str, striatum; WM, white matter; CTX, cortex. [file 1756-6606-4-35-S2.JPEG]

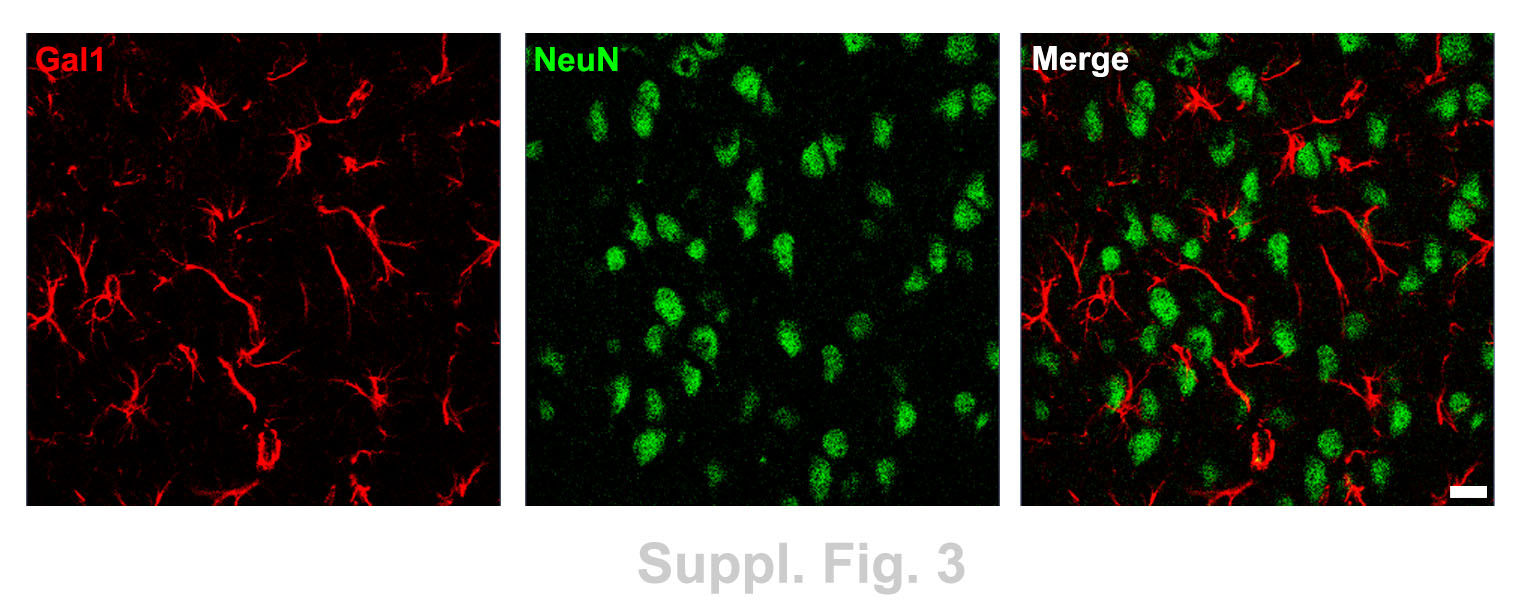

Supplement: Additional file 3 — Figure S3. The vast majority of Gal1-positive cells around the ischemic region were NeuN-negative. Representative image of Gal1(Red) and NeuN (Green) double-immunostaining around the ischemic region after brain ischemia (2 weeks after occlusion). (Scale bar: 25 μm). [file 1756-6606-4-35-S3.JPEG]

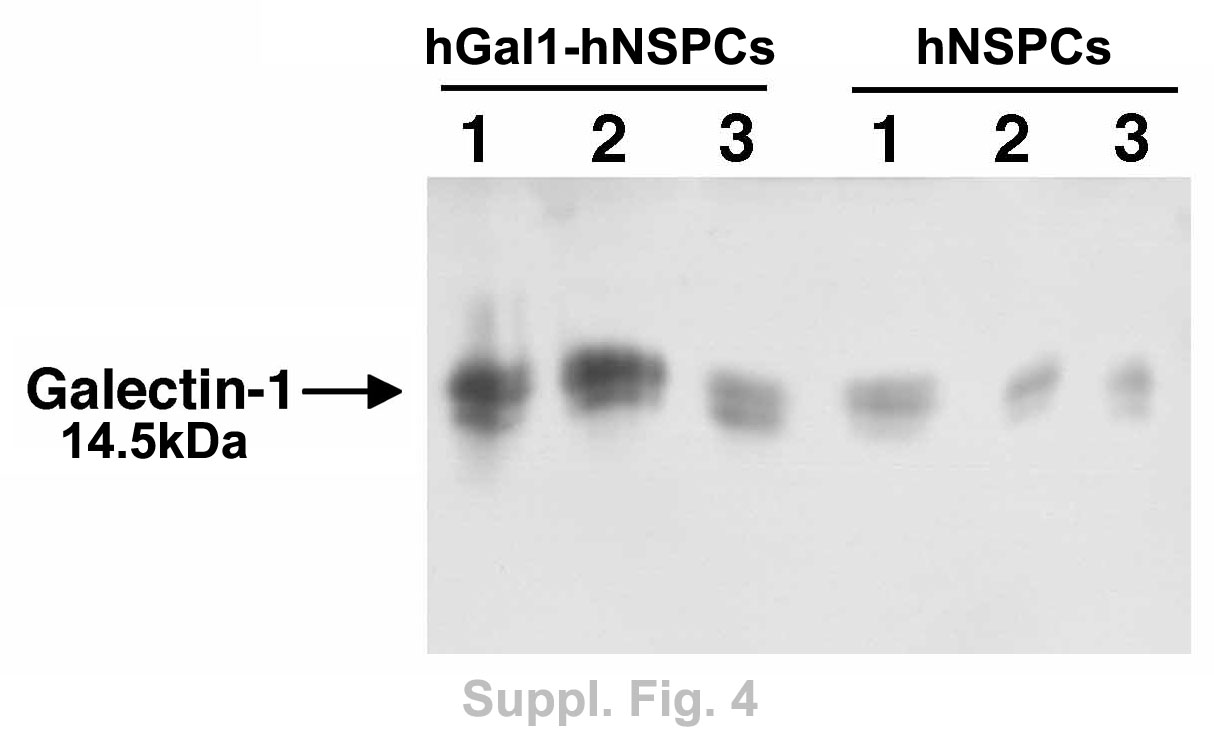

Supplement: Additional file 4 — Figure S4. Gal1 is expressed in the supernatant of hGal1-hNSPCs and hNSPCs cultures. The supernatant was isolated from hGal1-hNSPCs and hNSPCs cultures (n = 3 each), condensed, and then total volume of 300 μg protein from each culture was applied for western blot analysis using anti-hGal1-specific antibody. hGal1 (14.5 kDa) was detected in all of the supernatants. Note that the amount of hGal1 was increased in hGal1-hNSPCs culture compared with the hNSPCs culture. [file 1756-6606-4-35-S4.JPEG]

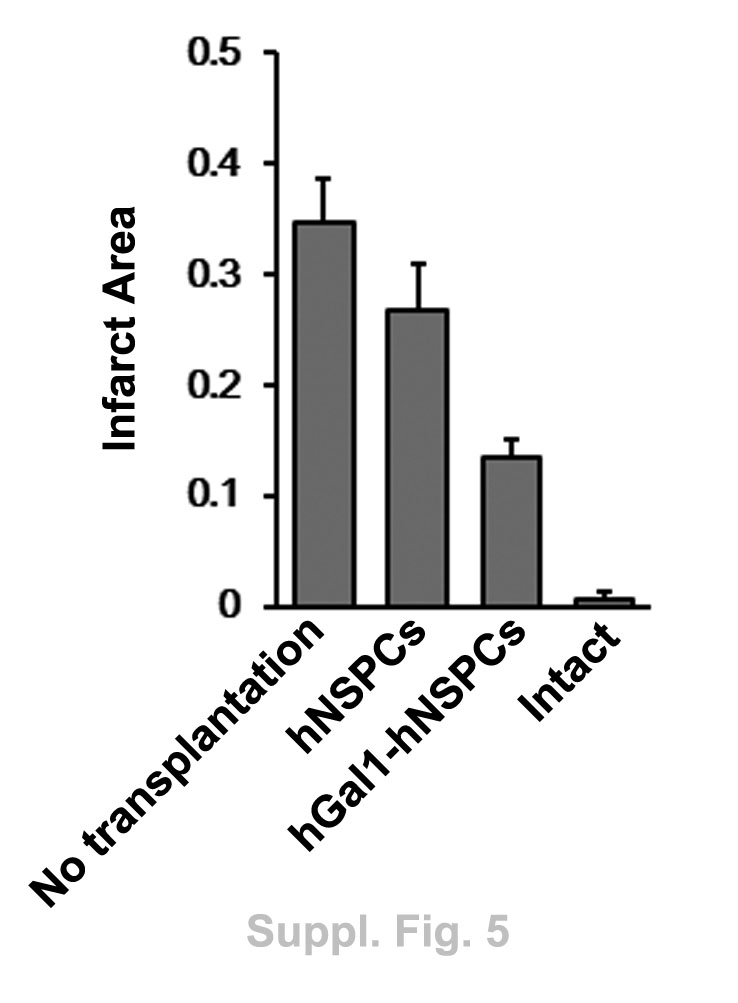

Supplement: Additional file 5 — Figure S5. Comparison of infarct area. Infarct area was calculated in the brains of no-transplantation, hNSCs, hGal1-hNSCs and intact groups. [file 1756-6606-4-35-S5.JPEG]
